# The duality of gaze: eyes extract and signal social information during sustained cooperative and competitive dyadic gaze

**DOI:** 10.3389/fpsyg.2015.01423

**Published:** 2015-09-23

**Authors:** Michelle Jarick, Alan Kingstone

**Affiliations:** ^1^Neurocognition of Attention and Perception Lab, Department of Psychology, MacEwan University, Edmonton, AB, Canada; ^2^Department of Psychology, University of British Columbia, Vancouver, BC, Canada

**Keywords:** gaze, attention, cooperation, competition, eye contact

## Abstract

In contrast to non-human primate eyes, which have a dark sclera surrounding a dark iris, human eyes have a white sclera that surrounds a dark iris. This high contrast morphology allows humans to determine quickly and easily where others are looking and infer what they are attending to. In recent years an enormous body of work has used photos and schematic images of faces to study these aspects of social attention, e.g., the selection of the eyes of others and the shift of attention to where those eyes are directed. However, evolutionary theory holds that humans did not develop a high contrast morphology simply to use the eyes of others as attentional cues; rather they sacrificed camouflage for communication, that is, to signal their thoughts and intentions to others. In the present study we demonstrate the importance of this by taking as our starting point the hypothesis that a cornerstone of non-verbal communication is the eye contact between individuals and the time that it is held. In a single simple study we show experimentally that the effect of eye contact can be quickly and profoundly altered merely by having participants, who had never met before, play a game in a cooperative or competitive manner. After the game participants were asked to make eye contact for a prolonged period of time (10 min). Those who had played the game cooperatively found this terribly difficult to do, repeatedly talking and breaking gaze. In contrast, those who had played the game competitively were able to stare quietly at each other for a sustained period. Collectively these data demonstrate that when looking at the eyes of a real person one both acquires and signals information to the other person. This duality of gaze is critical to non-verbal communication, with the nature of that communication shaped by the relationship between individuals, e.g., cooperative or competitive.

## Introduction

The human eye’s morphology is unique among primates in that it possesses a white sclera surrounding a darker iris and pupil. As a result of this high visual contrast, and unlike non-human primates, it is easy to determine where a human being is looking. One provocative proposal is that the high contrast polarity of the human eye is an evolutionary adaptation that occurred approximately six million years after the human and chimpanzee lineage split, and this singular morphological adaptation served as a catalyst for new forms of communication to emerge (Kobayashi and Kohshima, 1997). That is, unlike other primates, humans sacrificed camouflage of their looking behavior for communication. As a result we can determine quickly and quietly, and with remarkable fidelity, where someone else is looking, and this has a profound impact on our own behavior. For instance, much research suggests that the contrast polarity of the eyes can influence joint attention, such that human attention is oriented in the same direction as another’s gaze (Friesen and Kingstone, 1998; Driver et al., 1999). Moreover, Ricciardelli et al. (2009) have shown that reversing the contrast polarity of the eyes disrupts the perception and response to another’s gaze, supporting the importance of this factor in joint attention.

While a tremendous amount of research has been conducted on how humans discriminate and orient to the eyes of others, typically when those images of people are photos or schematic faces (e.g., Friesen and Kingstone, 1998; Hietanen and Leppanen, 2003), there has been a recent and growing appreciation in the field that the high contrast between iris and sclera does not exist only to support one’s ability to read the eyes of others as attentional cues. Rather it also serves to signal to others one’s internal states and intentions (see Risko et al., 2012; Laidlaw et al., in press, for reviews). The following recent studies illustrate this point.

In a natural situation between two individuals Wu et al. (2014) investigated if, and when, humans use gaze to signal information to other humans while eating. In a series of three experiments it was established that (1) there is a normative behavior to look away when someone begins to bite, (2) that people are more likely to look down at their food just before taking a bite, and pertinent to this paper, (3) when one person looks down signaling that a bite is forthcoming, the other person responds to that signal and looks away. These data suggest that natural gaze signaling occurs in social contexts (e.g., while sharing a meal), is read by another person, and can trigger a gaze response that is different from gaze following during joint attention. That is, the partner at the meal does not look down at the food or directly at the eater as a bite is about to be made but rather looks away in a manner that is consistent with the social norm (see also Wu et al., 2013).

More recently, Gobel et al. (2015) demonstrated that participants’ beliefs about social context could have a profound effect on the information that they signal with their eyes. They had participants watch videos of faces of higher or lower ranked people, while they, the participants, were filmed. The participants either believed that the recordings of their viewing behavior would later be seen by the people depicted in the videos or that no-one would see them. When participants believed that the recordings would later be seen by those depicted, they looked less at the eyes of the higher ranked people, and more at the eyes of the lower ranked individuals, suggesting that the participants used their gaze to signal information that was sensitive to social rank (e.g., Foulsham et al., 2010; Cheng et al., 2013).

Collectively, and critical to the aim of the present study, these recent studies suggest that natural real-time social attention between individuals is a two-way street, where each person signals as well as reads gaze information (Wu et al., 2014), and that the nature of this gaze signaling changes with the social context between individuals (Gobel et al., 2015). The present study combined these two ideas and put them to a direct test. We did this by requiring dyads, who did not know each other before taking part in the present study, to hold direct eye-gaze well beyond the natural period of a few seconds (Argyle and Dean, 1965). In addition, we manipulated the social context of the situation by having participants first play a competitive or a cooperative game. Our working hypothesis was that if making eye contact with another person brings into play the duality of eye gaze—that is, gaze serves to both read information from, and signal information to, another person—and that the nature of this gaze communication varies with social context (Wu et al., 2013), then requiring people to hold their eye gaze far beyond the comfort zone of a few seconds should serve to amplify the communication that is occurring between individuals to the point that it would be observable in their behavior alone.

Admittedly, this is a rather bold prediction, but it is grounded on the foundational ideas that eye gaze (as evidenced by its unique morphology) is an extraordinarily powerful and important visual stimulus to humans that supports communication between individuals. Furthermore, as the above data from Wu and Gobel suggest, the use of eye gaze is extremely sensitive to social context and the norms that reside within them. Indeed, social context has such a powerful force on looking behavior that when individuals who do not know each other are together in shared space there is a marked tendency to avoid looking at each other. This has been demonstrated recently on several occasions (Foulsham et al., 2011; Laidlaw et al., 2011; Gallup et al., 2012). For instance, Laidlaw et al. (2011) demonstrated that people sitting in a waiting room were more likely to look in the direction of a chair if it was empty than when it was occupied by a stranger.

In other words, there is good reason to think that people will find it extremely difficult to look at a stranger in the eye for a prolonged period of time. So much so that we hazard to guess that if the reader of this brief report imagines walking into a study, playing a game with a stranger, and then being asked to sit down beside this new partner and for the next 10 min to stare into her or his eyes while s/he stares deeply into theirs, that simply imagining this situation might cause the reader to feel some discomfort. One might also be able to imagine that the nature of the game that one first played with their partner, and the social context that it established, could have a tremendous impact on what one might feel is being communicated while looking into each other’s eyes. For example, if the game was cooperative in nature, then the communication might be positive and unifying, almost intimate, and one might try to break eye contact or talk about something neutral to reduce the intimacy being created. In contrast, if the game and social context with the partner was competitive in nature, then the dynamic might feel more like a staring contest.

Consistent with these proposals, research has shown that strangers who wish to limit the level of intimacy will reduce the degree to which they make eye contact (Argyle and Dean, 1965), while those who wish to portray dominance will engage in more eye contact (Exline et al., 1965). Therefore, we predicted that dyads in the cooperative group might try to limit their use of eye contact to keep the intimacy level at bay, while the dyads in the competitive group might keep eye contact to heighten their dominance. The null hypothesis was that this task would be easy and insensitive to any changes in social context primed by having the participants first play a short game. After all, the participants did not know the person they were partnered with, the preceding game, as we will show, involved simply working on puzzles, and the task itself “just” involved looking into the eyes of another person.

## Materials and Methods

### Participants

Forty-two undergraduate students participated (15 males, 27 females, mean age of 20 years). Participants were tested in pairs (21 dyads in total). One dyad admitted to having been in class together and were excluded from the analysis. All other participants reported being strangers and provided informed consent prior to participating. There were 10 cooperative dyads (7 males, 13 females; seven same-sex and three opposite-sex) and 11 competitive dyads (8 males, 14 females; seven same-sex and four opposite-sex). All participants gave informed consent before participating and the Research Ethics Boards approved the study procedures.

### Procedure

Dyads were randomly assigned to either the cooperative or competitive context. For the cooperative context, participants were asked to complete a series of Tangram puzzles together as a team, whereas for the competitive context each participant completed their own Tangram puzzle in a race against the other person. Participants had 5 min to complete as many Tangram puzzles as they could. Tangram puzzles are a type of dissection puzzle, composed of different geometric pieces that can be combined to form a broad range of different shapes and/or patterns. The task is to combine all the puzzle pieces to form the requested shape and/or pattern, then move onto the next requested shape/pattern, and so on.

**FIGURE 1:**
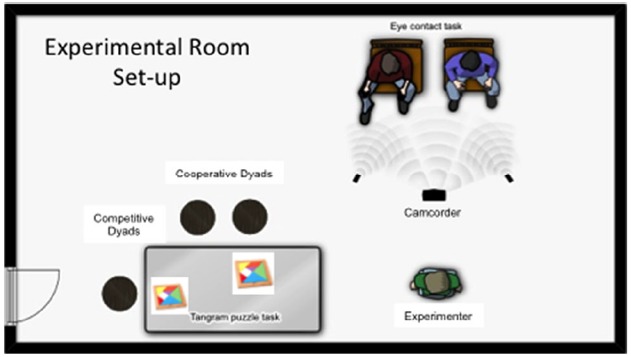
**Schematic representation of the experimental room set-up between the puzzle and eye contact phases of the experiment and where participants were situated during the cooperative and competitive contexts**.

All participants were seated at the same table, with cooperative dyads beside one another and competitive dyads at different sides of the table (see Figure 1, for a schematic of the set-up). Thus, all participants in the competitive context could see each the others’ progress, which was designed to add to the competitive nature of the situation. Consistent with the different nature of the games, all the dyads in the cooperative task engaged in conversation with one another while performing the task, typically with conversation about the task—its difficulty, what pieces should go where, etc.,—ongoing throughout the 5-min session. In contrast, it was unusual for the competitive dyads to talk with one another, and they never engaged in any helping cooperative behaviors, such as assisting the other individual with solving a puzzle. These observations provided us with a solid basis for believing that the two tasks had been successful in establishing different types of relationships between the two groups, i.e., cooperative or competitive. And while we do not have eye contact and speech data from the cooperative dyads, a recent paper by Ho et al. (2015) did track the eyes of dyads while they engaged in cooperative games, and they found that eye gaze is used to signal both the end and the beginning of a speaking turn. Specifically, a speaker will end his or her speaking turn with direct gaze at the listener, and the listener will then begin to speak while averting their gaze. Note that these data make the additional important point that both eye contact, and the breaking of eye contact, are important communicative social signals.

**FIGURE 2:**
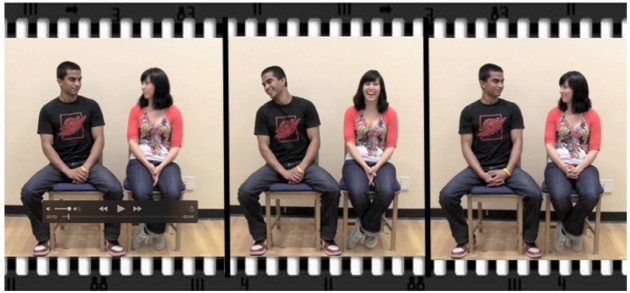
**Example of the eye contact phase of the experiment.** Participants were seated in close proximity, akin to sitting on a bus or next to someone in a classroom.

After the puzzle game, participants were asked to relocate to a different section of the room and sit next to one another (about one foot between them). They were instructed to make eye contact for as long as they could within a 10-min period and it was emphasized that they were not to “cheat,” e.g., by closing their eyes or looking at another part of the partner’s face. If they broke eye contact, they were to tell the experimenter and just start again until the 10-min had elapsed. There was no penalty for breaking eye contact (save for the fact that it extended the total time required to accumulate a total of 10 min of eye contact time) and the experimenter was very patient with participants when they did break eye contact. Participants had to stay still in their seats and only turned their head toward their partner to make eye contact. We reasoned that having participants sit side-by-side would maximize the physical proximity between them in a natural way (e.g., akin to sitting on a bus) and ensure that when their heads were turned they would be very close to one another (see Figure 2). Because a head turn of this nature is effortful, and as such there is no question that the act is anything but volitional, we reasoned that it would serve only to further enhance the gaze signal. These were the only limitations for participants and they were otherwise free to talk, smile, laugh, etc.

Eye contact was evaluated using three different sources. The first source was the participants themselves. They were explicitly instructed not to “cheat” and to self-report when they felt eye contact was broken. The second source was the experimenter. He was trained to watch participants and stop them if he detected a break in eye contact, e.g., a look elsewhere on the face of the participant’s partner. The third source was the video recorded using three HD Sony camcorders (two capturing the faces of each participant and one capturing the interaction of both participants). The video was analyzed offline (with 1080p resolution) by two independent coders (author MJ and a research assistant) who were blind to the cooperative and competitive conditions.

## Results

**FIGURE 3:**
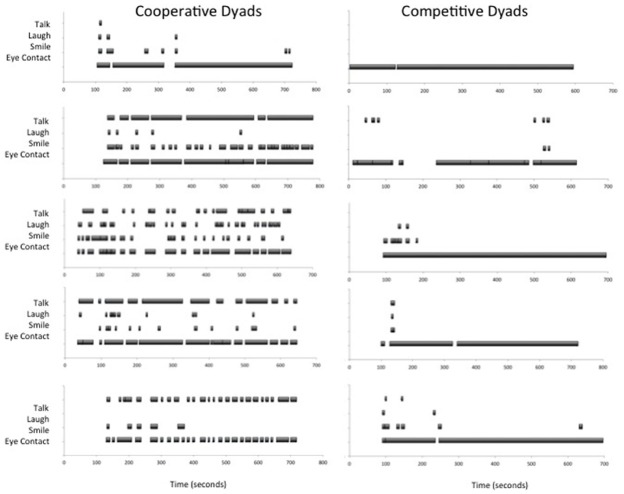
**Scarf plots representing both duration and frequency of participant behaviors as a function of time across the 10-min period**.

The videos were coded for the behavioral markers of gaze, smiling, laughing, and talking. The inter-rater reliability was high for the proportion of all behaviors recorded (*r* = 0.99 for eye contact, *r* = 0.82 for talking, *r* = 0.62 for smiling, and *r* = 0.82 for laughing). Figure 3 shows scarf plots representing the behavioral markers as a function of the 10-min period for five representative dyads in each group. Note that some dyads total time exceeds 10-min (600 s) due to the occurrence of spontaneous interruptions, e.g., a sneeze, the asking of a question, etc. The data analysis however is specific to the 10-min engaged in the task of trying to keep eye contact.

These scarf plots are presented to illustrate how qualitatively different the two types of dyads performed. The cooperative dyads general behavior, presented on the left of Figure 3, is punctuated by talking, laughing, smiling and repeated failures to maintain eye contact for sustained periods of time. In contrast, the competitive dyads presented on the right of Figure 3, rarely talk, laugh or even smile; and hold direct eye gaze with one another for remarkably long sustained periods of time, with a break in gaze clearly the exception rather than the rule. These patterns of behavior illustrate that the Tangram puzzle prime was a powerful manipulation in our study, and converge with the predicted outcomes of our study, i.e., that dyads in the cooperative group would find it difficult to sustain eye contact while the dyads in the competitive group would not. While talking is consistent with the positive social relationship between the dyads, many also casually reported that engaging in conversation helped them to make the eye contact experience less uncomfortable (i.e., less intimate). For instance, cooperative dyads might acknowledge that they should stop talking and focus on the task of keeping eye contact, but then within a few seconds of direct eye contact were back to conversing. Also consistent with this, the conversation topics tended to be non-intimate small-talk about school, work, extracurricular activities, etc. Those under the competitive social context, on the other hand, were able to sustain gaze and did not feel it necessary to talk, smile, or laugh.

**FIGURE 4:**
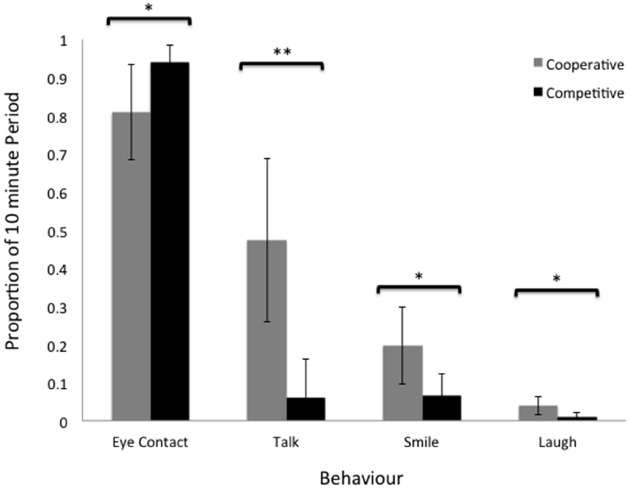
**Mean proportions of eye contact, talking, smiling, and laughing for both cooperative and competitive dyads.** Error bars represent the 95% confidence intervals and double asterisks “**” represent significance at the 0.01 level and single asterisk “*” represent significance at the 0.05 level.

In order to commit key aspects of these data to statistical analysis, the observed behaviors—eye contact, talk, smile, laugh—were averaged for each dyad (since the behaviors of each participant in the dyads co-occurred) and subjected to independent-sample one-tail *t*-tests with the proportion of time spent performing the behaviors as dependent variables. See Figure 4, for mean proportions across the two groups.

The results showed a significant difference between the groups in the proportion of the 10-min making eye contact [*t*(19) = 2.005, *p* = 0.029], the proportion of the time spent talking [*t*(19) = 3.56, *p* = 0.001], the proportion spent smiling [*t*(19) = 2.299, *p* = 0.016], and the proportion of time laughing [*t*(19) = 2.26, *p* = 0.018]. That is, the competitive group was able to keep eye contact for longer periods (*M* = 93.9% of the time) compared the cooperative group (*M* = 80.9% of the time), while the cooperative group talked significantly more (*M* = 47.3 vs. 5.9%), smiled significantly more (*M* = 19.7 vs. 6.6%), and laughed significantly more (*M* = 3.9 vs. 0.1%) compared to the competitive group.

As most of the dyads were of the same-sex pairs, reliable same- vs. opposite-sex comparisons could not be made. However, we did remove the opposite-sex pairs to evaluate the data for a mixed-sex bias and the results did not change. With the same-sex pairs all behavioral measures were significantly different across the conditions (all *p-values* < 0.05) showing the robustness of our effects.

## Discussion

In general, researchers have assumed that social attention in the real world can be studied by investigating how people attend to images of people (e.g., Friesen and Kingstone, 1998; Hietanen and Leppanen, 2003). Over the past few years, however, investigators have begun to make the argument that studying how people attend to mere representations of people is failing to capture a key aspect of social attention in the real world (Kingstone et al., 2003, 2008; Myllyneva and Hietanen, 2015). That is, we do not only look at other people simply to extract information about where they are looking. We also look at other people to signal to them information about ourselves, just as they look at us to signal information about themselves. This looking to others to extract information as well as signal information is what we refer to as the *duality of gaze*, although we hasten to add that this duality is not strictly limited to looks toward individuals, as looks away from people also serve an important communicative signal (e.g., Ho et al., 2015).

To date, the amount of evidence in support of this latter position has been limited, but what has been collected has been consistent with it. Some (but by no means all) of the evidence was touched on in the introduction to the present paper. For example, there is also work by Freeth et al. (2013) demonstrating that people answering questions from a live interviewer vs. a video recorded interviewer were sensitive to changes in eye contact only with the live interviewer. Similarly, Foulsham et al. (2011) has reported that people avert their gaze when approaching a real person vs. a video of that person. All these studies are predicated on the notion that there is a duality of gaze that exists in a live situation that is absent when faced with a video version. However, none directly test the idea that live direct gaze is communicative in nature. The present study does precisely that.

In a deceptively straightforward experiment we show that when people are required to make eye contact for a sustained period of time, the social relationship that has been primed between individuals dictates whether eye contact can be kept or not. When the social relationship was cooperative, eye contact was very difficult to sustain, and talking became very frequent, consistent with the notion that individuals find eye contact uncomfortable and reduce this discomfort by limiting the sending and receiving of (potentially intimate) gaze signals and distract themselves with conversation. An alternative, and not mutually exclusive possibility, is that participants are attempting to regulate their emotional arousal by breaking gaze. Future investigation will be required to resolve if one or both possibilities are being applied.

In contrast, when the relationship between the two participants has been primed to be competitive, participants were able to maintain direct eye gaze for longer stretches of time—far beyond what is normal—and they engaged in relatively little talking. This is consistent with the idea that within a competitive context, eye contact could be perceived as a portrayal of dominance and performed as a staring contest. Indeed, a few participants in the competitive condition spontaneously voiced the strategy of a staring contest.

In sum, this simple study stands as a singular, explicit and powerful demonstration that when two individuals make eye contact, their gaze serves a communicative function that is exquisitely sensitive to and shaped by small manipulations in their relationship. Just by asking participants to work together on a puzzle for 5 min, either cooperatively or competitively, can profoundly alter their ability to sit side by side and look each other in the eye for a period of time.

In addition to the theoretical implications of the present study, the current investigation raises two interesting methodological contributions as well. The first concerns the effectiveness of the Tangram game in priming a cooperative or competitive relationship between participants. This has not, to our knowledge, been demonstrated before and is therefore a potentially powerful tool for future social scientists wishing to manipulate the relationship between two or more individuals in a subtle but robust manner. Secondly, there is the staring task itself. It is not an understatement to say that the task of asking participants to stare at one another could be one of the most powerful quick tests for a researcher to use to determine the underlying nature of their relationship. If dyads have great difficulty keeping eye contact and indulge in talking with one another, then it will serve as an indicator that their relationship is a cooperative one. Conversely, if they have little difficulty making eye contact and fail to talk much, then one might infer that theirs is a more competitive one. That said, it is also important to note that at present we do not have a clear notion of what is the “baseline” performance on this task. While it is tempting to think that no puzzle task, or doing the puzzle task alone, will provide a baseline measure, this would merely leave the relationship between dyads free to vary as a function of whether the dyads found the eye contact task cooperative or competitive. Indeed there are many other social factors that may also modulate the nature of the eye contact task—such as the perceived attractiveness of the individuals in the dyads, their culture, their sexual orientation, and their social status—each of which will further complicate what is the “true baseline” performance.

In closing, and with the caveats above in place, it is perhaps worthwhile to indulge in a small degree of speculation about the behaviors we observed as a function of eye gaze and social context, and what factors may be found to be driving these behaviors after future investigation. With the clear acknowledgment then that what follows is speculation, it is generally assumed that eye contact signals interpersonal thoughts, attitudes, and intentions (e.g., Baron-Cohen et al., 1997), but little is known about if or how it does so during live social interactions. Some of the early researchers to study this phenomenon focused on how eye contact influenced the level of intimacy or dominance when performed at close distances (Argyle et al., 1973). For instance, researchers showed that individuals make more eye contact with people that they like and are attracted to (Exline et al., 1965). Another study reported that couples in love make more eye contact overall than couples that were not in love (Rubin, 1970). Compellingly, strangers have reported feelings of passionate love after spending only 2-min engaged in unbroken eye contact (Kellerman et al., 1989). To account for these effects of eye contact and intimacy, Argyle and Dean (1965) proposed that the level of intimacy between strangers could be maintained by balancing four factors: eye contact, proximity, topic of conversation, and smiling. For instance, if one wants to keep intimacy levels low, they should stand further apart, reduce eye contact, and talk about something banal such as the weather.

More recently, Ponkanen and Hietanen (2012) demonstrated that eye contact with a live individual causes a significant increase is nervous system arousal (galvanic skin response) and this was even more pronounced in response to a smiling face than a neutral face. Arousal is a physiological response to intimacy and according to Argyle and Dean’s initial proposal one could predict that arousal would show the greatest enhancement to eye contact of a smiling face in close proximity and engaging in a personal conversation.

Against this historical backdrop one might wish to speculate then that the cooperative dyads in the present study were already in a higher-than-normal intimate environment by simply sitting close in proximity to one another. Hence, the most direct way to reduce intimacy was to break eye contact, which is what we observed. However, because the task was to maintain eye contact, the other avenue was to engage in neutral conversation. This is also what we observed. The competitive dyads on the other hand, were close in proximity but primed to exert dominance. Thus their need to break eye contact or engage in idle conversation was relatively low, and hence the finding that for this group eye contact was sustained and talking was not.

## Conclusion

Here we showed experimentally that the effect of eye contact could be quickly and profoundly altered by the social context that was primed by a simple puzzle game. Those who had played the game cooperatively found eye contact terribly difficult to sustain and indulged in a great deal of talking, smiling and laughing. In contrast, those who had played the game competitively were able to stare quietly at each other for long periods with little smiling or laughing. These findings support our hypothesis that when looking at the eyes of a real person, one both acquires and signals information to the other person. This duality of gaze is critical to non-verbal communication, with the nature of that communication shaped by the relationship between individuals, i.e., cooperative or competitive.

### Conflict of Interest Statement

The authors declare that the research was conducted in the absence of any commercial or financial relationships that could be construed as a potential conflict of interest.
